# In Response

**DOI:** 10.1213/ANE.0000000000007539

**Published:** 2025-04-17

**Authors:** Kariem El-Boghdadly, Jaideep J. Pandit

**Affiliations:** Department of Anaesthesia, Guy’s and St Thomas’ NHS Foundation Trust, London, UK; Nuffield Department of Anaesthetics, Oxford University Hospitals NHS, Oxford, UK, jaideep.pandit@sjc.ox.ac.uk

We thank Higgs et al^1^ for their letter about our editorial,^2^ in which they emphasize their view that capnography over all other signs should be used to exclude esophageal intubation. We note that the authors make no comment on the tube tip in pharynx (TTIP) technique,^3^ which was the stimulus for our commentary. The study by Markham et al^3^ suggests that TTIP undermines the case that capnography is, in isolation, central to confirmation of tracheal intubation, because with TTIP there is an end-tidal capnography trace, yet the tube is not only outside the trachea, but at risk of migration into the esophagus. We would welcome Higgs et al comments on how TTIP impacts interpretation of capnography in this context.

Higgs et al criticize our citing of a report where supposed adherence to Project for Universal Management of the Airway (PUMA) guidance might have led practitioners to place overemphasis on, or too literally interpret the lack of capnography trace, causing removal of a correctly sited tube, and consequent patient death. The report we cited is sparse in detail, but the accompanying commentary clearly stated the link that “the tube was correctly sited, but due to the lack of a trace on the monitor, it was incorrectly diagnosed as being esophageal intubation. *This is a rare but catastrophic example of when ‘No Trace’ did not indicate ‘Wrong place’.”*^4^ Readers should interpret this independently but to us, the words in italics highlight the need to revisit the simple message that capnography should always override other lines of evidence as to tube position. We can all agree that any algorithm depends on monitors being checked and working.

We do not understand their statement that: “PUMA guidance is on (a) excluding esophageal intubation rather than confirming either (b) tracheal or (c) esophageal intubation.” Imagining a simple Venn diagram as an aid to propositional logic of the relationships between (a), (b) and (c) [our notation], will show how the two things (esophageal versus tracheal tube position) are mutually exclusive.^5^ So, “confirming tracheal intubation” (point b) automatically “excludes” (point a) “esophageal intubation” (point c). Our message is that tracheal intubation needs to be confirmed, as if it is not confirmed, then the tracheal tube may be either in the esophagus or in the pharynx.

Our suggested flowchart^2^ does not, as Higgs et al claim, enshrine confirmation bias but the opposite, as it emphasizes the need to reconcile findings from multiple sources (clinical, physiological and anatomical) with heightened concern where these do not align. Moreover, our approach captures the distinction between tube position (which can be correct or incorrect) versus lung ventilation (which can be effective or ineffective). Usually an incorrectly sited tube goes hand in hand with ineffective ventilation, but evidence from TTIP, shows that this is not always the case. Importantly, our suggested flowchart is not in fact contradictory to PUMA guidelines. Rather, it adds the important element of uncertainty that might be safer than a false certainty based on a single variable.

Higgs et al seem to place weight on national society endorsement for PUMA guidance, but this does not constitute scientific evidence. No guidance is perfect, and debate is the only way to improve and evolve guidelines, particularly in an area with a dearth of high-quality evidence and relying on inferential secondary data.^6,7^ We stress that both PUMA guidance and our suggested flowchart, likely need formal testing before either is widely adopted. In the meantime, we believe that dogma and undue emphasis on certainty might be associated with dangerous outcomes, and we therefore encourage clinicians to err on the side of caution, and to recognize uncertainty, as they do in so many other clinical judgements. The results of Markham et al on TTIP provide a stimulus for us to be less certain about a single variable (capnography) and instead use it in conjunction with a range of other potential variables to improve patient safety.


**Kariem El-Boghdadly, FRCA** *Department of Anaesthesia* *Guy’s and St Thomas’ NHS Foundation Trust* *London, UK*
**Jaideep J. Pandit, DPhil, FRCA** *Nuffield Department of Anaesthetics* *Oxford University Hospitals NHS* *Oxford, UK* *jaideep.pandit@sjc.ox.ac.uk*

